# Microparticles shed from multidrug resistant breast cancer cells provide a parallel survival pathway through immune evasion

**DOI:** 10.1186/s12885-017-3102-2

**Published:** 2017-02-06

**Authors:** Ritu Jaiswal, Michael S. Johnson, Deep Pokharel, S. Rajeev Krishnan, Mary Bebawy

**Affiliations:** 1 Discipline of Pharmacy, The Graduate School of Health, University of Technology Sydney, PO Box 123, Broadway, Sydney, NSW 2007 Australia; 2 iThree Institute, University of Technology Sydney, Sydney, NSW 2007 Australia

**Keywords:** Cancer, Extracellular vesicles, Immune evasion, Macrophage, Microparticles, Multidrug resistance, Cell engulfment

## Abstract

**Background:**

Breast cancer is the most frequently diagnosed cancer in women. Resident macrophages at distant sites provide a highly responsive and immunologically dynamic innate immune response against foreign infiltrates. Despite extensive characterization of the role of macrophages and other immune cells in malignant tissues, there is very little known about the mechanisms which facilitate metastatic breast cancer spread to distant sites of immunological integrity. The mechanisms by which a key healthy defense mechanism fails to protect distant sites from infiltration by metastatic cells in cancer patients remain undefined.

Breast tumors, typical of many tumor types, shed membrane vesicles called microparticles (MPs), ranging in size from 0.1–1 μm in diameter. MPs serve as vectors in the intercellular transfer of functional proteins and nucleic acids and in drug sequestration. In addition, MPs are also emerging to be important players in the evasion of cancer cell immune surveillance.

**Methods:**

A comparative analysis of effects of MPs isolated from human breast cancer cells and non-malignant human brain endothelial cells were examined on THP-1 derived macrophages in vitro. MP-mediated effects on cell phenotype and functionality was assessed by cytokine analysis, cell chemotaxis and phagocytosis, immunolabelling, flow cytometry and confocal imaging. Student’s *t*-test or a one-way analysis of variance (ANOVA) was used for comparison and statistical analysis.

**Results:**

In this paper we report on the discovery of a new cellular basis for immune evasion, which is mediated by breast cancer derived MPs. MPs shed from multidrug resistant (MDR) cells were shown to selectively polarize macrophage cells to a functionally incapacitated state and facilitate their engulfment by foreign cells.

**Conclusions:**

We propose this mechanism may serve to physically disrupt the inherent immune response prior to cancer cell colonization whilst releasing mediators required for the recruitment of distant immune cells. These findings introduce a new paradigm in cancer cell biology with significant implications in understanding breast cancer colonization at distant sites. Most importantly, this is also the first demonstration that MPs serve as conduits in a parallel pathway supporting the cellular survival of MDR cancer cells through immune evasion.

## Background

Breast cancer is the most frequently diagnosed cancer in women. In breast cancer, dissemination to distant organs is common. Resident macrophages at distant sites provide a highly responsive and immunologically dynamic innate immune response against foreign infiltrates [1, 2]. Immunosurveillance and the concept of tumour cell ‘foreignness’ was first proposed by Ehrlich in 1909. Immunosurveillance describes the processes by which cells of the immune system stringently search for, recognize and destroy foreign cells in the body. This stringent control fails with cancer progression and evasion of this immune response can occur through various mechanisms, including through reduced immune recognition, increased resistance to attack by immune cells or the development of an immunosuppressive tumour microenvironment [3].

Macrophages are phagocytic white blood cells of the innate immune system, which reside in all tissues and are central to the immune response. Macrophages maintain tissue integrity through their capacity to detect, engulf and destroy foreign cells. They can be activated by a variety of stimuli and polarize to functionally different phenotypes, including the classically activated (M1) and alternatively activated (M2) phenotypes [4]. Despite extensive characterization of the role of macrophages in malignant tissues, the mechanisms by which this key defense mechanism fails to protect distant sites from infiltration and colonization by metastatic cells in cancer remain undefined.

Breast tumours, typical of many tumour types, systemically shed membrane vesicles or extracellular vesicles [5] called microparticles (MPs), ranging in size from 0.1–1 μm in diameter [6]. MPs differ from cellular exosomes on the basis of size and cellular origin, with the latter originating from intracellular multivesicular bodies. MPs rather, arise from the ubiquitous process of plasma membrane blebbing [7]. Our previous work has shown that MPs provide a “non-genetic” basis for the acquisition, dissemination and dominance of deleterious cancer traits such as multidrug resistance (MDR) and enhanced metastatic capacity in cancer cells [8–10]. Specifically, we showed MPs (i) are shed in vast quantities in the context of malignancy [11]; (ii) serve as vectors in the intercellular transfer of functional resistance proteins and nucleic acids [8, 10, 12–14]; (iii) “re-template” the transcriptional landscape of recipient cells to ensure the acquisition of deleterious donor cell cancer traits [14, 15]; (iv) provide a reservoir for active and passive drug sequestration [16]; (v) confer onto recipient breast cancer cells an enhanced metastatic capacity [9] and, (vi) provide a tissue selective mechanism for the transfer of resistance in breast cancer [12, 13].

In addition, there are reports on the role of EVs derived from immune and non-immune cells in mechanisms contributing to the regulation of the immune response in inflammation, autoimmune diseases and cancer. B-cell derived EVs have been shown to stimulate T-cells directly, and antigen presenting cells indirectly, through the transfer of antigenic peptides (tumorigenic, pathogenic and B-cell receptor native antigens) [17–21]. The fusion of cancer cell-derived MPs with monocytes have been shown to inhibit monocyte differentiation [22, 23], whereas MPs bearing latent membrane protein can inhibit leukocyte proliferation [24]. Tumour-derived EVs have also been shown to contain Fas Ligand, which may induce apoptosis in activated antitumor cytotoxic T lymphocytes and decrease the cytotoxicity of natural killer cells [25].

Despite these studies, the role of EVs in the regulation of the immune response, specifically in the evasion of the immune response in the context of cancer, is an area of progressive investigation. There is nothing known about the role of MDR tumour-derived MPs in regulating the immune response. MDR describes the mechanism by which cells become cross resistant to a wide range of structurally and functionally unrelated molecules following the exposure to a single agent [26]. Synonymous with MDR is the overexpression of drug efflux transporters, the main players in drug detoxification pathways, of which P-glycoprotein (P-gp) is the main contributor in mammals [6, 27].

In this paper we report on the discovery of a new cellular pathway by which MDR breast cancer cells can functionally incapacitate and ultimately engulf macrophage cells through the transfer of MPs. This mechanism of macrophage priming through cancer cell derived MPs may serve as a cellular mechanism in establishing the pre-metastatic niche. This introduces a new paradigm in cancer cell biology with significant implications in immune evasion by cancer cells and the role of MPs in establishing the pre-metastatic niche. This is also the first demonstration that MPs serve as conduits in a parallel pathway supporting the cellular survival of MDR cancer cells through immune evasion.

## Methods

### Cell culture

The human monocytic leukemia cell line, THP1 was a kind gift from Dr Brian Oliver (University of Technology, Sydney, NSW, Australia). The human breast adenocarcinoma cell line, MCF-7/Dx was initially developed from the drug-sensitive human breast adenocarcinoma cell line, MCF-7 cells by incremental exposure to doxorubicin hydrochloride (DOX) and diplays a strong resistance to the drug and is multidrug resistant [15]. All three cell lines were maintained in RPMI 1640 growth medium (Sigma-Aldrich, NSW, Australia) supplemented with 10% (v/v) heat-inactivated fetal bovine serum (Life Technologies, Victoria, Australia) in the absence of antibiotics, under a humidified atmosphere at 37 °C and 5% CO_2_. A non-malignant immortalised human brain endothelial cell line hCMEC/D3, sequentially immortalized by lentiviral vector transduction with the catalytic subunit of human telomerase (hTERT) and SV40 large T antigen [28] was grown in EGM-2 medium (Lonza CC-3162) in T-175 flasks and maintained under the same conditions as described above. The MCF-7 cells were a kind gift from Dr Rosanna Supino (Istituto Nazionale per lo Studio e la Cura dei Tumori, Milan, Italy) and Dr Suzanne M. Cutts (La Trobe University, Victoria, Australia) and the hCMEC/D3 from Prof Georges E. R. Grau (The University of Sydney, NSW, Australia).

### Microparticle purification

MP’s were isolated from confluent MCF-7/Dx (Res) or MCF-7 (Sen) or hCMEC/D3 (D3) cells by differential centrifugation, as previously described [8, 15]. Briefly, culture supernatants were collected and centrifuged at 500 g for 5 min to pellet whole cells. The collected supernatant was re-centrifuged at 15,000 g for 1 h at 15 °C to pellet the MPs. The final pellet was resuspended in serum free RPMI 1640 media and centrifuged at 2000 g for 1 min to remove debris. The clear MP suspension was further centrifuged at 18,000 g for 30 min at 15 °C to pellet MPs. The isolated MP pellets were validated for typical MP characteristics of size and phosphatidylserine exposure using flow cytometer (FCM) (BD^TM^ LSR II, BD Biosciences) after V450 Annexin V (BD Biosciences) as described previously by us [8]. The MCF-7/Dx cells were selected for these studies as we have previously shown them to be highly metastatic and hence they provide a suitable in vitro model for metastatic breast cancer [9]. MPs isolated from MCF-7/Dx, MCF-7 or hCMEC/D3 cells are referred to as Res-MPs, Sen-MPs or D3-MPs respectively for simplicity [8, 15]. Total protein content of MPs was determined using the Quant-iT™ protein assay as per the manufacturer’s instructions (Life Technologies Australia).

### Differentiation of THP-1 cells to Macrophages

1x10^5^ THP-1 cells were differentiated into macrophages on a flat bottom 96 well plate in a total of 300 μL complete medium using 50 ng/mL phorbol 12-myristate 13-acetate [29] (Sigma-Aldrich), for 3 days. After the initial 3 days stimulus, the PMA containing media was removed; the cells were washed thrice and replaced with fresh complete media. This was followed by incubating the cells for a further 3 days. This allows for the enhancement of differentiation of the PMA treated cells [30]. The resulting macrophages displayed typical characteristics of increased auto-fluorescence, phagocytic activity and CD11b marker (BD Bioscinces) relative to THP-1 monocytes [30, 31]. The macrophages were stable in culture for at least 5 days post differentiation and all experiments described were conducted within this timeframe [30].

### Functional activity of THP-1 Macrophages

#### Phagocytic activity

1x10^5^ THP-1 macrophages with or without Res-MP, Sen-MP and D3-MP treatments for 4 h or 24 h were tested for phagocytic activity by overnight incubation with 1.5 μL of carboxylate-modified 2 μm diameter red fluorescent beads (Sigma-Aldrich) in a total of 300 μL culture media at 37 °C. Cells were harvested, washed twice with DPBS and analysed by flow cytometry. The number of latex beads ingested was calculated based on the percentage increase in PE channel with respect to the percentage in the PE channel of cells not incubated with beads.

#### Chemotaxis

The chemotactic effect was determined using transwell inserts (24-well, 6.5 mm insert, pore size 8 μm, Corning). Equal number of THP-1 macrophages treated with or without Res-MP, Sen-MP or D3-MP for 4 h were seeded on the apical chamber of the inserts in serum free media. Media with 10% FBS was added to the basal chamber as a chemoattractant. After 24 h incubation, the cells which had emerged from the basal side of the inserts were fixed and stained with 0.5% crystal violet in 20% methanol for 10 min.

### Microparticle treatment of Macrophages

THP-1 macrophages differentiated on a 96-well flat bottom plates were treated with 100 μg of Res-MPs, Sen-MPs or D3-MPs for 4 h in a total of 200 μL complete culture medium at 37 °C and 5% CO_2_. After 4 h, the cells were washed thrice with Dulbecco’s phosphate buffered saline (DPBS) (Sigma-Aldrich) to remove unbound MPs. Following MP treatment, cells were harvested with accutase solution (Sigma-Aldrich) for further analysis.

### Flow cytometry

The expression of ICAM-1 (CD54) and CD44 (Sigma-Aldrich) was assessed on the THP-1 macrophages before and after MP treatment. Cells were stained with 30 μL anti-ICAM-1 (1:100) or with or anti-CD44 (1:30) followed by Alexa Fluor 405 goat anti-mouse IgG (Life Technologies) (1:200) or with Alexa Fluor 647 goat anti-rabbit IgG (Life Technologies) (1:400). Samples were analysed by flow cytometry to assess the percentage cell surface expression.

### Cell internalization assays

#### Confocal microscopy

THP-1 macrophages and Sen or Res or D3 cells were harvested and stained with the CellTracker Green or CellTrace™ Far Red Cell Proliferation dye (Life Technologies) respectively for 45 min at 37^0^ C in serum-free RPMI 1640. Stained cells were washed twice in complete media, mixed (in a ratio of 1:1) and 10^5^ total cells seeded on coverslips placed in 6 well plates in 2 mL growth medium. The mixed cells were exposed to 50 μg Res-MP or Sen-MPs or D3-MPs and incubated for 24 h. Following incubation, the cells were washed thrice with PBS and fixed in 2% formaldehyde in PBS. Images were acquired using the 60x oil lens and 1.4 NA using the confocal laser scanning Nikon A1 microscope (Nikon). The z-series images were re-constructed using the Imaris software package (Bitplane AG, Zurich, Switzerland).

#### Flow cytometry

To quantitatively assess cell internalization, Sen or Res cells were labelled with 3 μM CFSE (carboxyfluorescein diacetate succinimidyl ester) dye (Stemcell Technologies, VIC, Australia) for 10 min at 37^0^ C in serum-free RPMI 1640. Labelling was stopped with complete media and the cells were washed twice prior to co-culture with macrophages. 5×10^4^ THP-1 macrophages were co-cultured (ratio of 1:1) with the CFSE labelled Sen or Res or D3 cells. 50 μg of Res-MP or Sen-MP or D3-MPs were added to the heterotypic cell cultures and following 24 h incubation cells were harvested and stained with a macrophage marker, APC conjugated anti-CD11b antibody (BD Biosciences). Samples were incubated for 30 min in the dark, washed twice in PBS and analysed for dual labels and single labels with the BD LSR Fortessa™ X-20 flow cytometer. The cells which were dual positive for both markers (CFSE-green channel and CD11b-red channel) represent those cells engulfed by macrophages. The remainder of the population comprises of macrophages alone, cells which have engulfed macrophages or cells alone. This was measured by the percentage drop in the population of each of these in their respective channels.

### SDS PAGE and western blot

Total cellular proteins were separated on 4–12% NuPAGE Bis-Tris gel (Life Technologies) before transferring to PVDF membrane (Pall Australia, VIC, Australia). The membrane was blocked, incubated with anti-Hyaluronic acid mAb (LS-C315053) (Sapphire Bioscience, NSW, Australia). Anti-β-actin (clone AC-74) (Sigma-Aldrich) was used as the internal control, followed by horseradish peroxidase-conjugated secondary antibody and subjected to enhanced chemiluminescence (Roche, VIC, Australia).

### Cytokine assays

For the cytokine array, 1x10^5^ THP-1 macrophages before and after Res-MP or Sen-MP or D3-MP treatment in 96 well plates were incubated for 18 h. Supernatants were collected and the protein levels of the cytokines IL-1β, IL-6, IL-10, TNF-α, IFN-γ and GM-CSF were determined by Luminex®, magnetic beads using the Milliplex Human High Sensitivity T Cell magnetic panel-6-Plex Kit (Millipore, NSW, Australia) and further validated using the Human Cytokines Multi-Analyte ELISArray Kit (SABiosciences, VIC, Australia) according to manufacturer’s instructions.

### Statistical analysis

All experiments were performed in triplicate. GraphPad Prism software was used to plot the data and either Student’s *t*-test or a one-way analysis of variance (ANOVA) was used for comparison and statistical analysis between the sample populations. *P* values less than 0.05 (*p* < 0.05) were accepted as statistically significant.

## Results

### MPs shed from malignant and non-malignant cells bind to macrophages

A panel of malignant and non-malignant cell lines were used as donor cells for MP isolation. The non-malignant, immortalised human brain endothelial cell line hCMEC/D3 [32] together with the human breast adenocarcinoma drug sensitive cell line (MCF-7 designated Sen cells for simplicity) and its MDR subline (MCF-7/Dx designated Res for simplicity) were used. MPs isolated from these cells were referred to as Sen-MP, Res-MP and D3-MP respectively [8, 15]. MPs were validated for typical characteristics of size and phosphatidylserine exposure as described previously by us [8, 14]. The MCF-7/Dx cells were selected for these studies as we have previously shown them to be highly metastatic and hence provide a suitable in vitro model for metastatic breast cancer [9]. This metastatic cell line overexpresses the multidrug efflux transporter, P-gp making them also a typical model for P-gp mediated MDR [15].

The THP-1 macrophage model was used in our studies due to its practicality as it provides us with a readily inducible and immortalised macrophage human cell line with validated similarities to native macrophages [33].

**Fig. 1:**
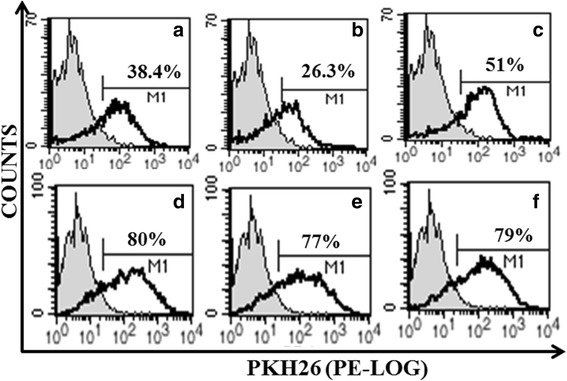
PKH-26 labelled MP binding to macrophage cells. 50 μg of MPs derived from malignant (**a, b**) and non-malignant cells (**c**) were labelled with PKH-26 followed by their co-culture with the THP-1 derived macrophages for 4 h. **a** 38.4% Res-MP, **b** 26.3% Sen-MP and **c** 51% D3-MPs were positive for PKH26 (*black open histogram*) relative to unstained control MPs (*gray filled histogram*). **d** 80%, **e** 77% and **f** 79% THP-1 macrophages were positive for PKH26 after co-culture with Res-MPs, Sen-MPs or D3-MPs respectively (*black open histogram*). Data represents a typical experiment (*n* = 3)

In establishing whether MPs shed from these cells bind to THP-1 macrophage cells, we used PKH26 (Life technologies, Victoria, Australia) (a red fluorescent amphiphilic cell linker dye) to label MPs as per our previous studies [12]. PKH26 irreversibly intercalates between membrane lipids without affecting MP viability, allowing for the identification of labelled MPs among heterogeneous populations by flow cytometry (FCM) [8]. 38, 26, and 51% of the isolated Res-MPs, Sen-MPs and D3-MPs stained positive for PKH labelling, respectively after overnight leaching as analysed by FCM (Fig. 1a–c). Following a 4 h co-culture of the PKH26 labelled MPs with THP-1 macrophages, 77–80% of the macrophages detected positive for PKH26 fluorescence (black open histogram) (Fig. 1d–f). These results confirm that the MPs derived from both malignant and non-malignant cells readily bind to macrophage cells establishing a capacity for heterotypic cell interactions.

### MPs modulate the release of pro-inflammatory cytokines from macrophages

Macrophages are highly plastic and can be polarized to a classically activated (M1) secreting high levels of pro-inflammatory cytokines or alternatively activated (M2) state secreting anti-inflammatory cytokines, depending on their environment [4]. It is unclear as to which state (s) macrophages adopt in the context of metastatic breast cancer, particularly following exposure to breast cancer derived MPs.

**Fig. 2:**
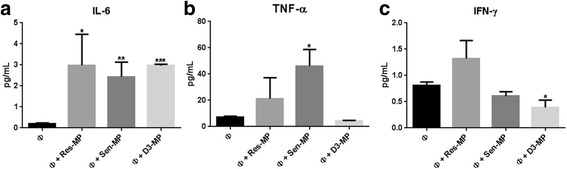
MPs increase the release of IL-6, TNF-α and INF-γ in THP-1 macrophages. THP-1 macrophage cells were co-cultured with 100 μg Res-MPs, Sen-MPs or D3-MPs for 4 h. Supernatants were collected and analysed using the Milliplex Human High Sensitivity T Cell magnetic panel-6-Plex Kit (Millipore, NSW, Australia) using luminex bead technology. **a** IL-6, **b**, IFN-γ and **c** TNF-α levels were analysed and mean ± SEM of at least 3 independent experiments conducted in duplicate are shown. Student’s unpaired two tailed *T*-test was used *P < 0.05, **P < 0.01, ***P < 0.001

To determine this, cytokine release in the cell supernatant prior to and following MP exposure was tested using the Milliplex Human High Sensitivity T Cell magnetic panel- 6-Plex Kit (Millipore, NSW, Australia) and further validated using the Human Cytokines Multi-Analyte ELISArray Kit (SABiosciences, VIC, Australia). Consistent with THP-1 macrophages being mature macrophages with cytokine secreting capacity [29] we detected basal levels of both pro-inflammatory and anti-inflammatory (data not shown) cytokines in the absence of stimuli. In the presence of MPs we observed a significant change from basal levels only for pro-inflammatory cytokines IL-6, TNF-α and INF-γ (Fig. 2). We observed a significant increase in the release of IL-6 by all three MPs (Fig. 2a). The exposure of macrophages to Sen-MPs resulted in elevated TNF-α level (Fig. 2b) whereas exposure to D3-MPs resulted in a significantly suppressed INF-γ response (Fig. 2c). We observed no effect by MPs on GM-CSF, IL-10 and IL-1β levels (data not shown). These results demonstrate that MPs shed from both malignant and non-malignant cells induce the release of pro-inflammatory cytokines following their transfer to macrophage cells.

### MPs impair macrophage functionality

The effect of MPs on macrophage phagocytic and chemotactic capacity was next examined.

#### Phagocytosis

**Fig. 3:**
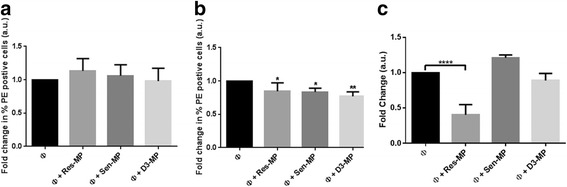
Phagocytic and chemotactic incapacity in macrophages following MP exposure. THP-1 macrophages were assessed after 24 h for their ability to phagocytise PE-labelled beads by flow cytometry following (**a**) 4 h MP co-culture or (**b**) 24 h MP co-culture. **c** THP-1 macrophages were assessed at 24 h following a 4 h MP co-culture for their ability to migrate through a transwell membrane (8 μm). Data was analysed using the BD LSR II, flow cytometer. Data represents mean ± SEM values of three independent experiments. Student’s unpaired two tailed *T*-test with Welch’s correction was used for A and B and One way Annova used for C. *P < 0.05, **P < 0.01 and ****P < 0.0001

In assessing phagocytic activity, THP-1 macrophages were exposed to carboxylate-modified red fluorescent beads (2 μm in diameter) (Sigma-Aldrich) prior to and following their exposure to malignant Res-MP and Sen-MPs as well as the non-malignant D3-MPs (Fig. 3a–b). Phagocytic capacity was assessed by flow cytometry and calculated based on the percentage increase in the PE channel for the cells exposed to beads relative to cells without exposure to beads. The phagocytic activity of THP-1 macrophage cells to latex beads over a 24 h period was assessed following either a 4 h or 24 h MP exposure (Fig. 3a and b). We observed no significant effect for a 4 h MP exposure (Fig. 3a). We observed a slight but significant decrease in the phagocytosis of latex beads by THP-1 macrophages following exposure to all MPs (Fig. 3b).

#### Chemotaxis

The effect of MPs on chemotaxis was assessed using a transwell migration assay as previously described by us [9]. We observed a rapid and significant 63% reduction in chemotaxis in macrophages following a 4 h exposure to Res-MPs only (Fig. 3c). There was no significant effect observed on chemotaxis following exposure to either Sen-MP or D3-MP (Fig. 3c). These results demonstrate that THP-1 macrophages fail to display the functional characteristic of chemotaxis after Res-MP exposure despite residing in an activated pro-inflammatory state.

### MDR breast cancer derived MPs stimulate the engulfment of THP-1 macrophages by breast cancer cells

**Fig. 4:**
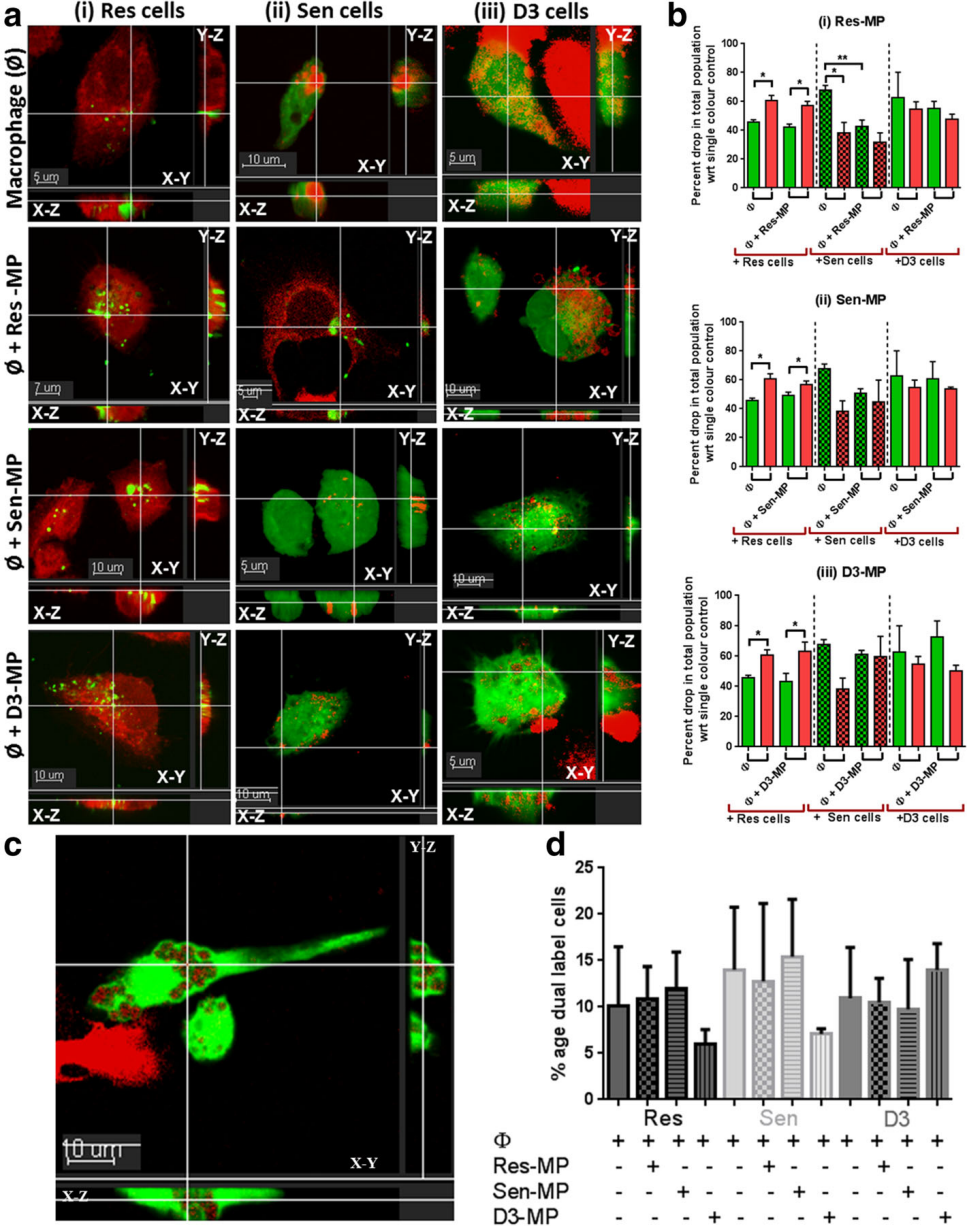
Macrophage engulfment by invading cells following exposure to resistant MP. **a** Fluorescence images show macrophages co-cultured with cells for 24 h +/− MPs. (**i**) Res cells labelled with Cell Trace Far Red dye internalise macrophages (Ø) labelled with the CellTracker Green dye in the presence and absence of all MPs. (**ii**) Sen cells (*red*) engulf macrophages following co-culture with Res-MPs only and (**iii**) D3 cells (non-malignant) (*red*) are engulfed by macrophages (*green*). Images are slice views of confocal z-series and show cell internalization. Data represents a typical experiment. Scale bar as indicated (**b**) Flow cytometric quantitation of cell engulfment by macrophages. Co-culture of APC conjugated anti-CD11b antibody labelled macrophages with CFSE labelled sensitive (Sen) or resistant (Res) or D3 cells +/− (**i**) Res-MPs, (**ii**) Sen-MPs, or (**iii**) D3-MPs. Data represents mean ± SEM (*n* = 3). Student’s unpaired two tailed *T*-test used *P < 0.05 and **P < 0.01. Phagocytosis of cells by macrophages. **c** Confocal image of the phagocytosis of MDR acquired Sen cell (following Res-MP exposure) by macrophages. Scale bar as indicated. Representative image shown. **d** Flow cytometric quantitation of phagocytosis. APC conjugated anti-CD11b antibody labelled macrophages were co-cultured with CFSE labelled Sen cells or Res cells and D3 cells +/− MPs. Macrophage engulfed cells are positive for both CFSE and CD11b. Data represents mean ± SEM values of three independent experiments

Our findings of loss of macrophage functionality led us to investigate the effects of MPs on macrophage ability to engulf heterotypic cells. We used confocal microscopy to examine the phagocytic capacity of CellTracker Green labelled macrophages in the presence of labelled cells using Cell Trace Far Red. We observed differential behaviour by macrophage cells towards different cell types prior to and following MP exposure (Fig. 4). Specifically, we observed that the resistant cells (MCF-7/Dx) were not engulfed by macrophages, contrary to that observed for the sensitive cells and the D3 cells (Fig. 4). Rather, the resistant cells displayed a remarkable capacity to engulf the THP-1 macrophages under all conditions (Fig. 4Ai). When the drug sensitive cells were co-cultured with Res-MPs we observed that these cells could now engulf the macrophages in a similar manner to the donor MCF-7/Dx cells (Fig. 4Aii). The exposure of Res-MPs to the Sen cells and to the THP-1 macrophage cells simultaneously confers MDR to the breast cancer cell and functional incapacity to the macrophage cells. This exposure facilitates the engulfment of macrophages by cancer cells. This result was not observed for the non-malignant cells. The D3 cells in all cases were engulfed by macrophages (Fig. 4Aiii). These results demonstrate that macrophage engulfment occurs only by MDR cells or cancer cells that had acquired MDR following the transfer of Res-MP cargo.

To quantitate these observations, we analysed our samples using flow cytometry. Macrophages were labelled with an APC-conjugated anti-CD11b antibody which detects the macrophage specific marker CD11b on the surface of macrophage cells. The malignant and non-malignant cells were labelled using the membrane dye CFSE. Those cells which were dual positive for both markers represent cells engulfed by macrophages on the basis of accessibility to the external CD11b, in absence of cell permeabilisation (Fig. 4C–D). Phagocytosis by macrophages was shown to represent 5–15% of the total cell population studied (Fig. 4D). The remainder of the population comprised of macrophages alone, cells which have engulfed macrophages or cells alone. To distinguish between these subpopulations, we measured the percentage drop in the population of each of these based on their respective labels. Again, consistent with our confocal findings we observed a significant drop in the macrophage population when they were co-cultured with both the resistant cells and following co-culture with Res-MPs and sensitive cells (Fig. 4Bi), consistent with their engulfment. Again these results provide support to our above findings and demonstrate that MDR cells, or cells that have acquired MDR have the capacity to engulf macrophages.

### Drug resistant breast cancer derived MPs mediate CD44 dependent clustering of macrophages

**Fig. 5:**
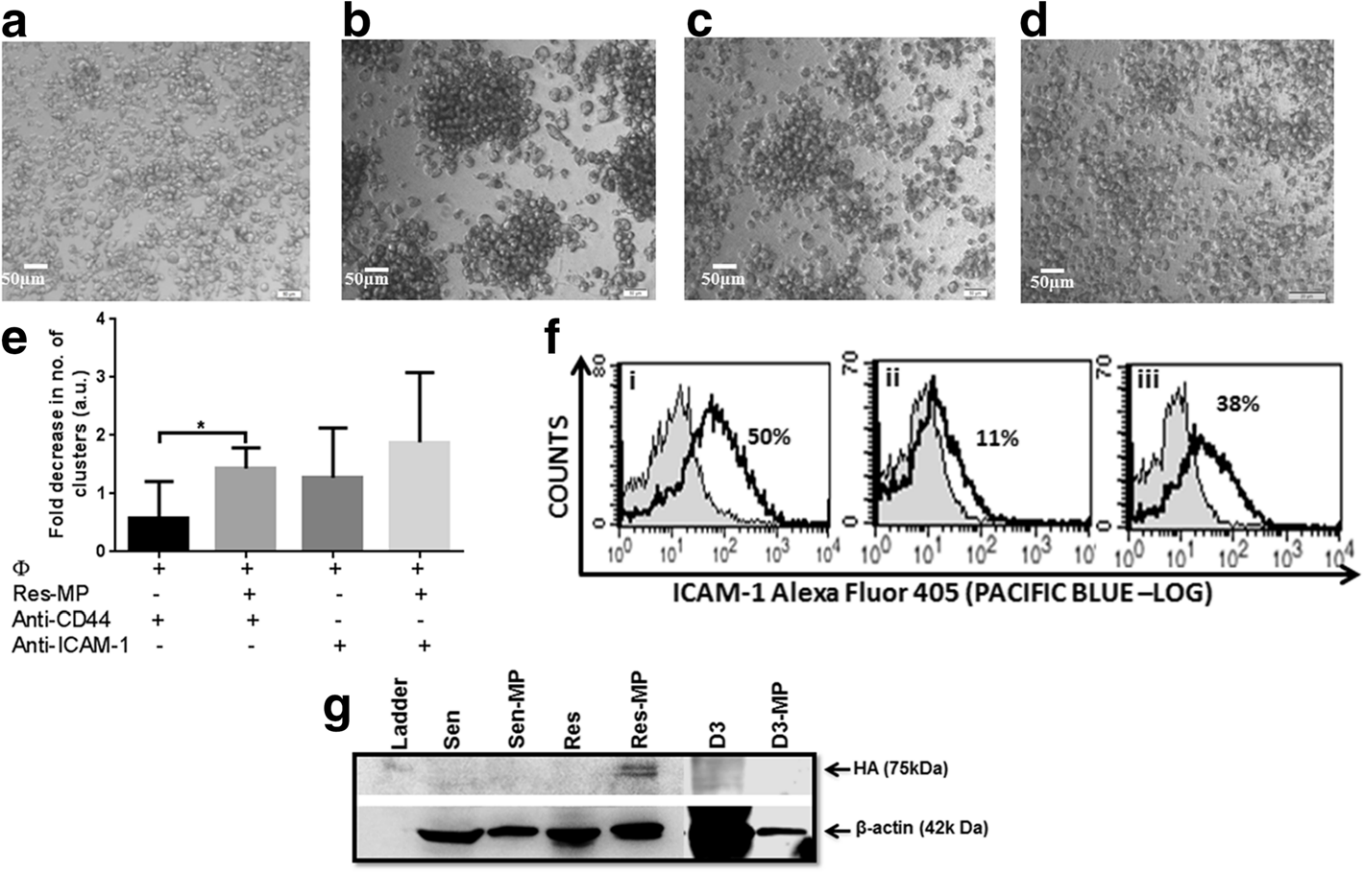
Drug resistant breast cancer derived MPs induce CD44 dependent clustering of macrophage cells. Cluster formation in macrophage cells grown in a monolayer (**a**) prior to and (**b**–**d**) following Res-MP co-culture for 24 h, **c** in the presence of anti-CD44 or (**d**) in the presence anti-ICAM-1 antibody. Images were acquired under a 10× magnification. Representative images shown. **e** Graphical representation of macrophage clustering with Res-MP. Data represents mean ± SEM values of three independent experiments. The student’s unpaired two tailed *T*-test with Welch’s correction was used for statistical analysis. *P < 0.05, **P < 0.01 and ****P < 0.0001. **f** Res-MPs induce expression of ICAM-1 in macrophage cells. (**i**) 50% of breast cancer-derived Res-MPs are positive for ICAM-1. Macrophage cells following 4 h co-culture with Res-MP show an increase in ICAM-1 expression from  (**ii**) 11% to (**iii**) 38% as determined by cell surface immunolabelling and flow cytometric detection. . Data is representative of a typical experiment. **g** Res-MPs selectively package hylauronic acid (HA). 30–50 μg lysates of the malignant drug sensitive breast adenocarcinoma cells (Sen), its drug resistant counterpart cells (Res) and the non-malignant D3 cells (D3) as well as MPs derived from them Sen-MP, Res-MP and D3-MP respectively were analysed by Western Blot analysis. The presence of HA was detected in the Res-MP only but not in the parental donor cell, the non-malignant cell neither their MPs. β-actin was used as an internal loading control. Representative data shown (*n* = 3)

It has been shown that the process of cell internalization is triggered by cell detachment [34]. We likewise observed in the THP-1 macrophages following MP exposure an increased capacity for cell aggregation and loss of surface adhesion to the supporting matrix of the cell culture plate (Fig. 5A). We observed a significant increase in the number of cell clusters and aggregates in the Res-MP co-cultured macrophages at least 24 h after co-culture (Fig. 5B) relative to macrophages alone or the Sen-MPs and D3-MPs co-cultures (Fig. 5A–E). Specifically, large raspberry like clusters were observed only in the presence of Res-MPs relative to macrophages alone or when co-cultured with Sen-MP and D3-MPs (Fig. 5A–E). We also observed a large number of detached cells in the supernatant culture media in macrophages co-cultured with Res-MPs relative to the untreated controls (data not shown). We confirm that almost 90% of cells in the supernatant were viable after co-culture and the aggregation was not the result of cell death (data not shown).

ICAM-1 or intracellular adhesion molecule 1 also known as CD54 is typically expressed on both endothelial and immune cells, including macrophages and is involved in cell to cell adhesion. In addition, the higher expression of ICAM-1 in mature dendritic cells (DC) derived EV cargo has been reported to exhibit enhanced T-cell binding and APC-T cell stimulatory function [35]. To examine the mechanism contributing to the observed clustering of macrophage cells in the presence of Res-MPs, we first examined the role of ICAM-1. Using flow cytometric analysis following direct immunolabelling for ICAM-1 we observed that 50% of the Res-MPs and 11% of macrophages express ICAM-1 respectively (Fig. 5Fi–ii). We observed a significant 2.4 fold increase in ICAM-1 surface expression on macrophages following Res-MP co-culture (Fig. 5Fiii). In the presence of an ICAM-1 neutralising antibody (monoclonal anti-ICAM-1) (Sigma Aldrich) (1:30 dilution) we observed no significant reduction in cell aggregation and clustering (Fig. 5D–E). This finding supports an alternative mechanism contributing to the observed cell aggregation.

We previously demonstrated using proteomic analysis that Res-MPs selectively package a number of proteins including P-gp and CD44 [13]. The ligation of CD44 on immune cells by hyaluronic acid (HA) has also been shown by numerous investigators to induce monocyte-T-cell aggregation [36, 37]. CD44 also has a role in cell to cell adhesion and is significantly expressed on the surface of macrophages [38], hence, we used a neutralising antibody (monoclonal anti-CD44) (Abcam) (1:30 dilution) on the macrophages and again assessed effects on cell aggregation after 24 h MP co-culture. We observed a significant 1.4 fold decrease in cell aggregation in the presence of a CD44 neutralizing antibody when Res-MPs were co-cultured with macrophages (Fig. 5C and E).

As activation of CD44 occurs through the binding of its endogenous ligand hyaluronic acid (HA) we probed the MPs for the presence of HA by Western Blot analysis. We again observed a selective packaging of HA in Res-MPs only and not in the Sen-MP, D3-MP or their donor cells (Fig. 5G). This finding demonstrates that unlike Sen-MPs or D3-MPs, Res-MPs selectively package HA in their cargo as they do its receptor CD44 [12, 13]. We confirmed by confocal microscopy and flow cytometric analysis using the anti-CD44 polyclonal antibody (Sigma-Aldrich) that 88% THP-1 macrophages express CD44 (data not shown).

These results demonstrate that aggregation and subsequent loss of surface adhesion of macrophages by Res-MPs is likely, if not in part, mediated by HA activation of CD44 expressed on the surface of macrophages following exposure to HA enriched Res-MPs.

## Discussion

Macrophages are highly plastic and can be polarized to a classically activated (M1) or alternatively activated (M2) state, depending on their environment [4]. M1 macrophages secrete high levels of pro-inflammatory cytokines, express high levels of MHC I and MHC II antigens, secrete complement factors and express high levels of nitric oxide synthase [39]. Conversely, M2 macrophages express the scavenger receptor, the mannose receptor and IL-10, which facilitate tumour progression amongst other things [40]. There is however nothing known about the activation state of macrophages neither in the context of metastatic breast cancer nor how MPs shed from malignant MDR cells interact with and regulate macrophage functionality.

We show that MPs shed from both malignant and non-malignant cells bind to human macrophage cells in vitro establishing a capacity for heterotypic cell interactions (Fig. 1). MPs, distinct from exosomes, are membrane vesicles which bud from the surface of cells, including breast cancer cells [14, 16]. Pioneering work by us has revealed multiple roles for MPs in cancer cell biology [8–11, 13–16]. Briefly, our published studies have shown that cancer derived MPs can bind readily to both homotypic and heterotypic cells to induce changes to the recipient cell transcriptome and phenotype to reflect that which is observed in the donor cell [8–10, 14, 15].

The binding of malignant (Sen-MP and Res-MPs) and non-malignant MPs (D3-MPs) to macrophage cells results in the polarisation of these cells towards a pro-inflammatory state. The increase in pro-inflammatory cytokine release by macrophages following exposure to EVs is supported by the findings of Chow et al., [41] who showed that exosomes released from breast cancer cells induced the transcription of pro-inflammatory cytokine proteins in macrophage cells. Our data shows for the first time that MPs induce pro-inflammatory cytokine secretion by macrophage cells.

The pro-inflammatory cytokine response is an essential part of macrophage functionality and at first instance would suggest an active immune response following exposure to cancer derived antigens in the form of the MP cargo. However, after further examination we show that THP-1 macrophages fail to display the typical functional characteristics of phagocytosis and chemotaxis following exposure to Res-MPs. Interestingly, the Sen-MPs and non-malignant D3-MPs only impaired phagocytosis and not chemotaxis in the macrophages. This differential effect could be attributed to the selective packaging of MP cargo in the Res-MPs relative to the Sen-MP or D3-MPs.

The induced functional incapacity of the macrophages by Res-MPs was followed by macrophage engulfment by Res cells contrary to that observed with Sen cells and D3 cells. When the Sen cells were co-cultured with Res-MPs however, we observed that these cells could now also engulf the macrophage cells in a similar manner to the resistant donor cells. These findings are interesting and support our earlier studies that demonstrated that breast cancer derived Res-MPs were tissue selective in the transfer of their MDR cargo to only malignant cells [12] and that the transfer of MPs resulted in the acquisition and dominance of the donor cell traits in recipient cell populations [14, 15]. Furthermore, we also previously demonstrated that the transfer of MDR cargo to drug sensitive breast cancer cells, imparted on these cells a capacity for increased migration and invasion [9], capabilities required for colonisation to distant sites and ultimatly the engulfment of macrophage cells. This cell in cell interaction can provide resistant tumour cells a nutrient source to sustain cell proliferation and growth [42] and also a mechanism for immune evasion by the metastatic cancer cell.

Cell in cell interactions are triggered by cell detachment [34]. The Res-MPs induced increased cell aggregation, loss of surface adhesion and detachment of the macrophages relative to Sen-MPs and D3-MPs (Fig. 5A–D), requirements of cell engulfment. In previous studies we have shown the selective packaging of CD44 within Res-MPs [12, 13]. We also show these same MPs to selectively package the CD44 ligand, HA.

The ligation of CD44 on immune cells by HA has also been shown by numerous investigators to induce monocyte-T-cell aggregation [36, 37]. The binding of HA to alveolar macrophages via CD44 elicits the expression of pro-inflammatory cytokines as well as plays a role in cell-cell adhesion [43]. Indeed, macrophage aggregation and detachment appeared to be dependent on the presence of CD44 on the surface of macrophages following Res-MPs co-culture. Our findings demonstrate that aggregation and subsequent loss of surface adhesion of macrophages by Res-MPs is likely to be mediated by HA activation of CD44 expressed on the surface of macrophages following exposure to HA enriched Res-MPs.

In relation to the loss of the chemotactic functionality observed in the macrophages following Res-MP co-culture, we propose that this is not attributed to macrophage aggregation and detachment. Previous studies have shown that high HA levels inhibit the chemotactic activity of polymorphonuclear leukocytes [44]. This suggests that chemotactic inactivity in macrophages in our study, may be attributed to the selectively packaged HA in the Res-MPs and not a consequence of physical resistance attributed to cell clustering and detachment.

Through our observation we suggest that the macrophages are incapacitated via Res-MPs and primed towards a pro-inflammatory state. This pro-inflammatory state may contribute to signalling for the recruitment of secondary immune cells to the malignant site. When macrophages are unable to maintain immunological integrity, macrophages recruit other cells of the innate immune response in a supportive capacity [45]. The recruitment of these secondary immune cells, facilitate the establishment of the malignant niche within the secondary site. Known as tumour associated macrophages (TAMs), these invading cells comprise up to 80% of the tumour cell mass [46, 47] and there is a strong correlation between the extent of TAM infiltration and poor prognosis [48]. TAMs originate from blood monocytes that are recruited from the peripheral circulation into, predominantly, the necrotic tumour core. In the tumour they constitute a distinct macrophage population that mediates cancer cell extravasation, establishment and growth [49, 50]. Meanwhile, breast cancer cells cannibalise the activated macrophages by inducing a loss of functional capacity, increased clustering, aggregation and detachment effectively escaping immune surveillance.

**Fig. 6:**
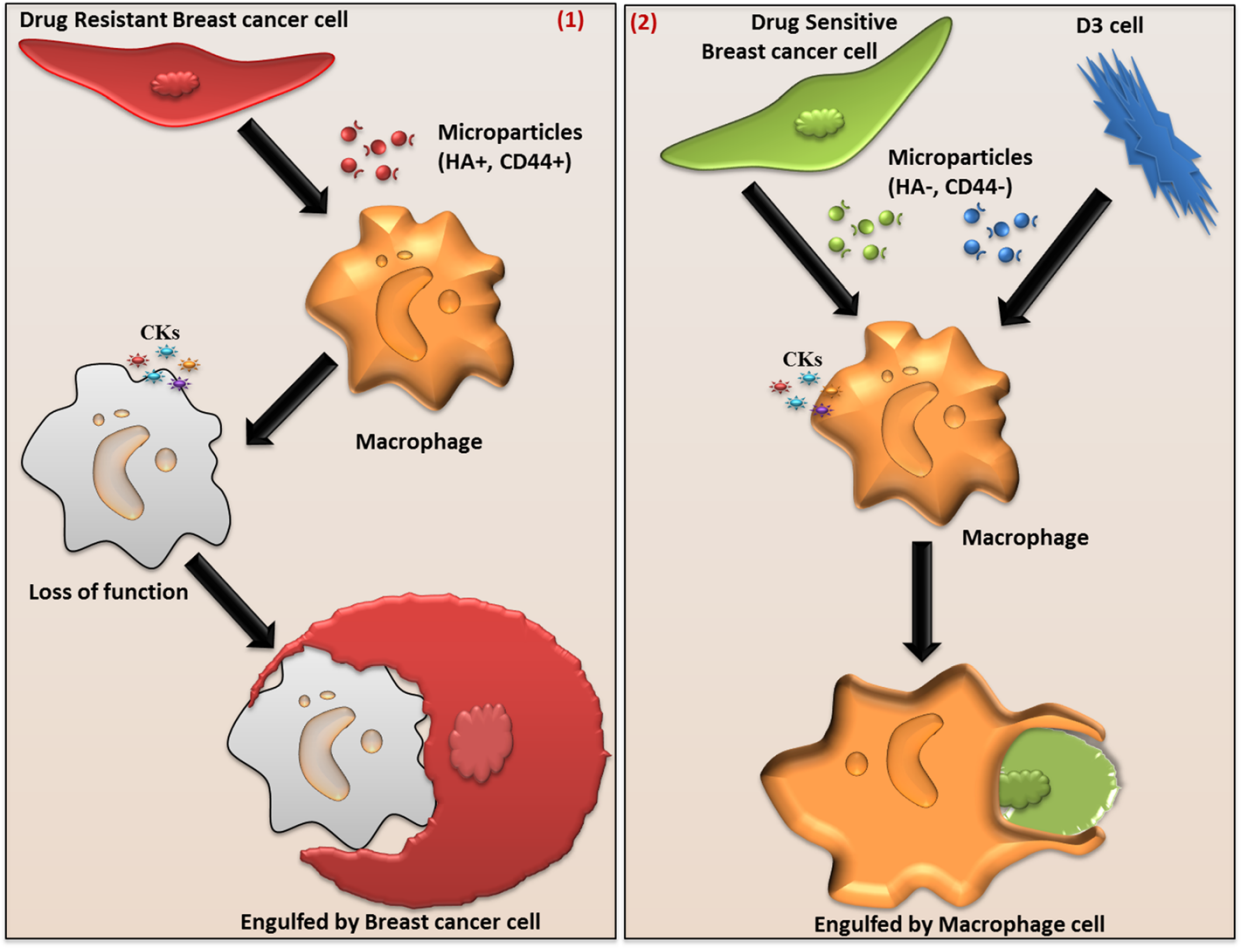
MPs shed from drug resistant cells mediate evasion of macrophage immunity: Overview of pathway: (1) MPs derived from resistant cancer cells bind to macrophage, stimulate a pro-inflammatory state (releasing cytokines-CKs), induce impaired macrophage chemotaxis and induce their engulfment by resistant or acquired resistant breast cancer cells. We propose this occurs through the Res-MP cargo which selectively packages P-gp, CD44 and HA relative to the (2) Sen-MP and D3-MPs

In summary we describe a novel pathway for tumour cell immune evasion and demonstrate that MDR breast cancer derived MPs have a (1) heterotypic interaction with the THP-1 macrophages, (2) activate the release of pro-inflammatory cytokines, (3) impair macrophage functionality and (4) stimulate the engulfment of THP-1 macrophages by MDR breast cancer cells (Fig. 6). Based on these findings we identify and propose a novel immune evasion pathway mediated by Res-MPs which ultimately leads to macrophage incapacity and engulfment to maintaining tumour resistance and survival (Fig. 6).

## Conclusions

In conclusion, MDR breast cancer derived MPs have a remarkable capacity to alter the phenotype and functionality of immune cells and in doing so can facilitate their destruction through engulfment. This failure to protect distant sites from the foreign infiltration by metastatic breast cancer cells may have a role in providing a permissive environment for secondary tumour colonisation. Given that metastatic disease is unresponsive to both conventional and emerging therapeutics, there is a need to explore strategies inherent to the immune arsenal as an adjunct or alternative therapeutic strategy. In achieving this, the immediate objective is to uncover the molecular basis for immune dysfunction at the metastatic site.

## Abbreviations

EV: Extracellular vesicles
MDR: Multidrug Resistance
MPs: Microparticles
TAMs: Tumour associated macrophages
